# Modeling aspects of the language of life through transfer-learning protein sequences

**DOI:** 10.1186/s12859-019-3220-8

**Published:** 2019-12-17

**Authors:** Michael Heinzinger, Ahmed Elnaggar, Yu Wang, Christian Dallago, Dmitrii Nechaev, Florian Matthes, Burkhard Rost

**Affiliations:** 1 Department of Informatics, Bioinformatics & Computational Biology - i12, TUM (Technical University of Munich), Boltzmannstr. 3, 85748 Garching/Munich, Germany; 2 TUM Graduate School, Center of Doctoral Studies in Informatics and its Applications (CeDoSIA), Boltzmannstr. 11, 85748 Garching, Germany; 3 Leibniz Supercomputing Centre, Boltzmannstr. 1, 85748 Garching/Munich, Germany; 4 TUM Department of Informatics, Software Engineering and Business Information Systems, Boltzmannstr. 1, 85748 Garching/Munich, Germany; 5 Institute for Advanced Study (TUM-IAS), Lichtenbergstr. 2a, 85748 Garching/Munich, Germany; 6 TUM School of Life Sciences Weihenstephan (WZW), Alte Akademie 8, Freising, Germany; 7 Department of Biochemistry and Molecular Biophysics & New York Consortium on Membrane Protein Structure (NYCOMPS), Columbia University, 701 West, 168th Street, New York, NY 10032 USA

**Keywords:** Machine Learning, Language Modeling, Sequence Embedding, Secondary structure prediction, Localization prediction, Transfer Learning, Deep Learning

## Abstract

**Background:**

Predicting protein function and structure from sequence is one important challenge for computational biology. For 26 years, most state-of-the-art approaches combined machine learning and evolutionary information. However, for some applications retrieving related proteins is becoming too time-consuming. Additionally, evolutionary information is less powerful for small families, e.g. for proteins from the *Dark Proteome*. Both these problems are addressed by the new methodology introduced here.

**Results:**

We introduced a novel way to represent protein sequences as continuous vectors (*embeddings*) by using the language model ELMo taken from natural language processing. By modeling protein sequences, ELMo effectively captured the biophysical properties of the language of life from unlabeled big data (UniRef50). We refer to these new embeddings as *SeqVec* (*Seq*uence-to-*Vec*tor) and demonstrate their effectiveness by training simple neural networks for two different tasks. At the per-residue level, secondary structure (Q3 = 79% ± 1, Q8 = 68% ± 1) and regions with intrinsic disorder (MCC = 0.59 ± 0.03) were predicted significantly better than through one-hot encoding or through Word2vec-like approaches. At the per-protein level, subcellular localization was predicted in ten classes (Q10 = 68% ± 1) and membrane-bound were distinguished from water-soluble proteins (Q2 = 87% ± 1). Although *SeqVec* embeddings generated the best predictions from single sequences, no solution improved over the best existing method using evolutionary information. Nevertheless, our approach improved over some popular methods using evolutionary information and for some proteins even did beat the best. Thus, they prove to condense the underlying principles of protein sequences. Overall, the important novelty is speed: where the lightning-fast *HHblits* needed on average about two minutes to generate the evolutionary information for a target protein, *SeqVec* created embeddings on average in 0.03 s. As this speed-up is independent of the size of growing sequence databases, *SeqVec* provides a highly scalable approach for the analysis of big data in proteomics, i.e. microbiome or metaproteome analysis.

**Conclusion:**

Transfer-learning succeeded to extract information from unlabeled sequence databases relevant for various protein prediction tasks. SeqVec modeled the language of life, namely the principles underlying protein sequences better than any features suggested by textbooks and prediction methods. The exception is evolutionary information, however, that information is not available on the level of a single sequence.

## Background

The combination of evolutionary information (from Multiple Sequence Alignments – MSA) and Machine Learning/Artificial Intelligence (standard feed-forward artificial neural networks – ANN) completely changed protein secondary structure prediction [1–3]. The concept was quickly taken up [4–8] and predictions improved even more with larger families increasing evolutionary information through diversity [9, 10]. The idea was applied to other tasks, including the prediction of transmembrane regions [11–13], solvent accessibility [14], residue flexibility (B-values) [15, 16], inter-residue contacts [17] and protein disorder [15, 18–20]. Later, automatic methods predicting aspects of protein function improved by combining evolutionary information and machine learning, including predictions of subcellular localization (aka cellular compartment or CC in GO [21, 22]), protein interaction sites [23–25], and the effects of sequence variation upon function [26, 27]. Arguably, the most important breakthrough for protein structure prediction over the last decade was a more efficient way of using evolutionary couplings [28–31].

Although evolutionary information has increasingly improved prediction methods, it is also becoming increasingly costly. As sequencing becomes cheaper, the number of bio-sequence databases grow faster than computing power. For instance, the number of UniProt entries is now more than doubling every two years [32]. An all-against-all comparison executed to build up profiles of evolutionary information squares this number: every two years the job increases 4-fold while computer power grows less than 2-fold. Consequently, methods as fast as PSI-BLAST [33] have to be replaced by faster solutions such as HHblits [34]. Even its latest version HHblits3 [35] still needs several minutes to search UniRef50 (subset of UniProt) for a single query protein. The next step up in speed such as MMSeqs2 [36] appear to cope with the challenge at the expense of increasing hardware requirements while databases keep growing. However, even these solutions might eventually lose the battle against the speedup of sequencing. Analyzing data sets involving millions of proteins, i.e. samples of the human gut microbiota or metagenomic samples, have already become a major challenge [35]. Secondly, evolutionary information is still missing for some proteins, e.g. for proteins with substantial intrinsically disordered regions [15, 37, 38], or the entire *Dark Proteome* [39] full of proteins that are less-well studied but important for function [40].

Here, we propose a novel embedding of protein sequences that replaces the explicit search for evolutionary related proteins by an implicit transfer of biophysical information derived from large, unlabeled sequence data (here UniRef50). We adopted a method that has been revolutionizing Natural Language Processing (NLP), namely the bi-directional language model ELMo (Embeddings from Language Models) [41]. In NLP, ELMo is trained on unlabeled text-corpora such as Wikipedia to predict the most probable next word in a sentence, given all previous words in this sentence. By learning a probability distribution for sentences, these models autonomously develop a notion for syntax and semantics of language. The trained vector representations (embeddings) are contextualized, i.e. the embeddings of a given word depend on its context. This has the advantage that two identical words can have different embeddings, depending on the words surrounding them. In contrast to previous non-contextualized approaches such as word2vec [42, 43], this allows to take the ambiguous meaning of words into account.

We hypothesized that the ELMo concept could be applied to model protein sequences. Three main challenges arose. (1) Proteins range from about 30 to 33,000 residues, a much larger range than for the average English sentence extending over 15–30 words [44], and even more extreme than notable literary exceptions such as James Joyce’s Ulysses (1922) with almost 4000 words in a sentence. Longer proteins require more GPU memory and the underlying models (so-called LSTMs: Long Short-Term Memory networks [45]) have only a limited capability to remember long-range dependencies. (2) Proteins mostly use 20 standard amino acids, 100,000 times less tokens than in the English language. Smaller vocabularies might be problematic if protein sequences encode a similar complexity as sentences. (3) We found UniRef50 to contain almost ten times more tokens (9.5 billion amino acids) than the largest existing NLP corpus (1 billion words). Simply put: Wikipedia is roughly ten times larger than Webster’s Third New International Dictionary and the entire UniProt is over ten times larger than Wikipedia. As a result, larger models might be required to absorb the information in biological databases.

We trained ELMo on UniRef50 and assessed the predictive power of the embeddings by application to tasks on two levels: per-residue (word-level) and per-protein (sentence-level). For the per-residue prediction task, we predicted secondary structure and long intrinsic disorder. For the per-protein prediction task, we predicted subcellular localization and trained a classifier distinguishing between membrane-bound and water-soluble proteins. We used publicly available data sets from two recent methods that achieved break-through performance through Deep Learning, namely NetSurfP-2.0 for secondary structure [46] and DeepLoc for localization [47]. We compared the performance of the *SeqVec* embeddings to state-of-the-art methods using evolutionary information, and also to a popular embedding tool for protein sequences originating from the Word2vec approach, namely *ProtVec* [42]. Notably, while *ProtVec* captures local information, it loses information on sequence ordering, and the resulting residue embeddings are insensitive to their context (non-contextualized), i.e. the same word results in the same embedding regardless of the specific context.

Understanding a language typically implies to understand most typical constructs convened in that language. Modeling a language in a computer can have many meanings, spanning from the automatic understanding of the semantic of languages, to parsing some underlying rules of a language (e.g. syntax). Arguably, proteins are the most important machinery of life. Protein sequence largely determines protein structure, which somehow determines protein function [48]. Thus, the expression of the language of life are essentially protein sequences. Understanding those sequences implies to predict protein structure from sequence. Despite recent successes [49, 50], this is still not possible for all proteins. However, the novel approach introduced here succeeds to model protein sequences in the sense that it implicitly extracts grammar-like principles (as embeddings) which are much more successful in predicting aspects of protein structure and function than any of the biophysical features previously used to condensate expert knowledge of protein folding, or any other previously tried simple encoding of protein sequences.

## Results

### Modeling protein sequences through SeqVec embeddings

*SeqVec*, our ELMo-based implementation, was trained for three weeks on 5 Nvidia Titan GPUs with 12 GB memory each. The model was trained until its *perplexity* (uncertainty when predicting the next token) converged at around 10.5 (Additional file 1: Figure S1). Training and testing were not split due to technical limitations (incl. CPU/GPU). ELMo was designed to reduce the risk of overfitting by sharing weights between forward and backward LSTMs and by using dropout. The model had about 93 M (mega/million) free parameters compared to the 9.6G (giga/billion) tokens to predict leading to a ratio of samples/free parameter below 1/100, the best our group has ever experienced in a prediction task. Similar approaches have shown that even todays largest models (750 M free parameters) are not able to overfit on a large corpus (250 M protein sequences) [51].

### SeqVec embeddings appeared robust

When training ELMo on SWISS-PROT (0.5 M sequences), we obtained less useful models, i.e. the subsequent prediction methods based on those embeddings were less accurate. Training on UniRef50 (33 M sequences) gave significantly better results in subsequent supervised prediction tasks, and we observed similar results when using different hyperparameters. For instance, increasing the number of LSTM layers in ELMo (from two to four) gave a small, non-significant improvement. As the expansion of 2 to 4 layers roughly doubled time for training and retrieving embeddings, we decided to trade speed for insignificant improvement and continued with the faster two-layer ELMo architecture. Computational limitations hindered us from fully completeing the modelling of UniRef90 (100 million sequences). Nevertheless, after four weeks of training, the models neither appeared to be better nor significantly worse than those for UniRef50. Users of the embeddings need to be aware that every time a new ELMo model is trained, the downstream supervised prediction method needs to be retrained in the following sense. Assume we transfer-learn UniRef50 through SeqVec1, then use SeqVec1 to machine learn DeepSeqVec1 for a supervised task (e.g. localization prediction). In a later iteration, we redo the transfer learning with different hyperparameters to obtain SeqVec2. For any given sequence, the embeddings of SeqVec2 will differ from those of SeqVec1, as a result, passing embeddings derived from SeqVec2 to DeepSeqVec1 will not provide meaningful predictions.

### Per-residue performance high, not highest

**Fig. 1:**
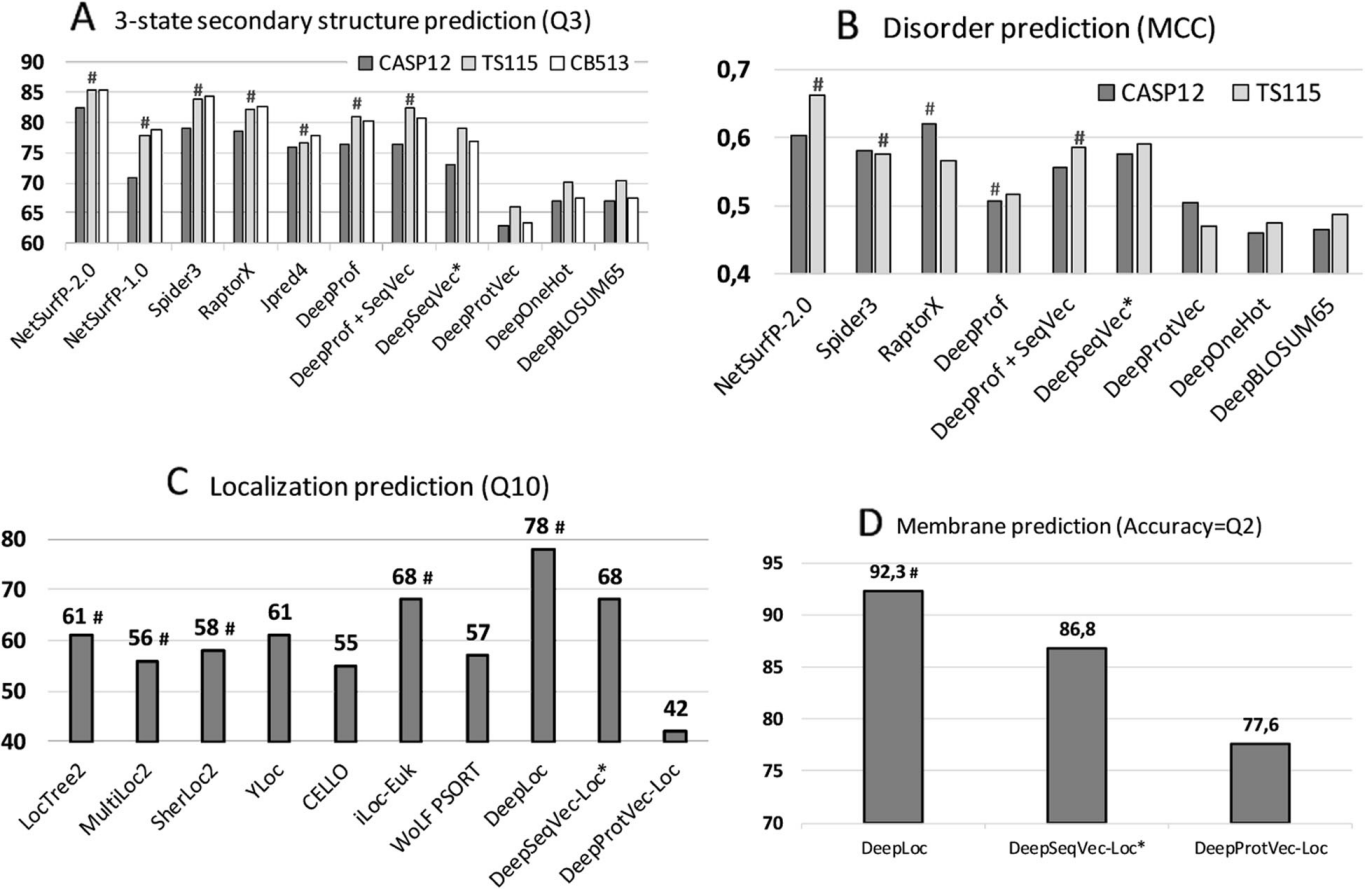
Performance comparisons. The predictive power of the ELMo-based SeqVec embeddings was assessed for per-residue (upper row) and per-protein (lower row) prediction tasks. Methods using evolutionary information are highlighted by hashes above the bars. Approaches using only the proposed *SeqVec* embeddings are highlighted by stars after the method name. Panel **A** used three different data sets (CASP12, TS115, CB513) to compare three-state secondary structure prediction (y-axis: Q3; all DeepX developed here to test simple deep networks on top of the encodings tested; DeepProf used evolutionary information). Panel **B** compared predictions of intrinsically disordered residues on two data sets (CASP12, TS115; y-axis: MCC). Panel **C** compared per-protein predictions for subcellular localization between top methods (numbers for Q10 taken from DeepLoc [47]) and embeddings based on single sequences (Word2vec-like *ProtVec* [42] and our ELMo-based *SeqVec*). Panel **D**: the same data set was used to assess the predictive power of SeqVec for the classification of a protein into membrane-bound and water-soluble

NetSurfP-2.0 feeds HHblits or MMseqs2 profiles into advanced combinations of Deep Learning architectures [46] to predict secondary structure, reaching a three-state per-residue accuracy Q3 of 82–85% (lower value: small, partially non-redundant CASP12 set, upper value: larger, more redundant TS115 and CB513 sets; Table 1, Fig. 1; several contenders such as *Spider3* and *RaptorX* reach within three standard errors). All six methods developed by us fell short of reaching this mark, both methods not using evolutionary information/profiles (DeepSeqVec, DeepProtVec, DeepOneHot, DeepBLOSUM65), but also those that did use profiles (*DeepProf*, DeepProf+SeqVec, Fig. 1a, Table 1). The logic in our acronyms was as follows (Methods): “*Prof*” implied using profiles (evolutionary information), *SeqVec* (Sequence-to-Vector) described using pre-trained ELMo embeddings, “Deep” before the method name suggested applying a simple deep learning method trained on particular prediction tasks using SeqVec embeddings only (DeepSeqVec), profiles without (DeepProf) or with embeddings (DeepProf+SeqVec), or other simple encoding schema (ProtVec, OneHot or sparse encoding, or BLOSUM65). When comparing methods that use only single protein sequences as input (DeepSeqVec, DeepProtVec, DeepOneHot, DeepBLOSUM65; all white in Table 1), the new method introduced here, *SeqVec* outperformed others not using profiles by three standard errors (*P*-value< 0.01; Q3: 5–10 percentage points, Q8: 5–13 percentage points, MCC: 0.07–0.12, Table 1). Using a context-independent language model derived from the Word2vec approach, namely DeepProtVec was worse by 10 percentage points (almost six standard errors). On the other hand, our implementation of evolutionary information (DeepProf using HHblits profiles) remained about 4–6 percentage points below NetSurfP-2.0 (Q3 = 76–81%, Fig. 1, Table 1). Depending on the test set, using *SeqVec* embeddings instead of evolutionary information (DeepSeqVec: Fig. 1a, Table 1) remained 2–3 percentage points below that mark (Q3 = 73–79%, Fig. 1a, Table 1). Using both evolutionary information and *SeqVec* embeddings (DeepProf+SeqVec) improved over both, but still did not reach the top (Q3 = 77–82%). In fact, the ELMo embeddings alone (DeepSeqVec) did not surpass any of the best methods using evolutionary information tested on the same data set (Fig. 1a).

**Table 1:** Per-residue predictions: secondary structure and disorder

*Data*	*Prediction task*	*Secondary structure*		*Disorder*	
	*Method*	*Q3 (%)*	*Q8 (%)*	*MCC*	*FPR*
*CASP12*	*NetSurfP-2.0 (hhblits)*^*a,b*^	**82.4**	**71.1**	0.604	**0.011**
	*NetSurfP-1.0*^*a,b*^	70.9	–	–	–
	*Spider3*^*a,b*^	79.1	–	0.582	0.026
	*RaptorX*^*a,b*^	78.6	66.1	**0.621**	0.045
	*Jpred4*^*a,b*^	76.0	–	–	–
	*DeepSeqVec*	73.1 ± 1.3	61.2 ± 1.6	0.575 ± 0.075	0.026 ± 0.008
	*DeepProf*^*b*^	76.4 ± 2.0	62.7 ± 2.2	0.506 ± 0.057	0.022 ± 0.009
	*DeepProf + SeqVec*^*b*^	76.5 ± 1.5	64.1 ± 1.5	0.556 ± 0.080	0.022 ± 0.008
	*DeepProtVec*	62.8 ± 1.7	50.5 ± 2.4	0.505 ± 0.064	0.016 ± 0.006
	*DeepOneHot*	67.1 ± 1.6	54.2 ± 2.1	0.461 ± 0.064	0.012 ± 0.005
	*DeepBLOSUM65*	67.0 ± 1.6	54.5 ± 2.0	0.465 ± 0.065	0.012 ± 0.005
*TS115*	*NetSurfP-2.0 (hhblits)*^*a,b*^	**85.3**	**74.4**	**0.663**	**0.006**
	*NetSurfP-1.0*^*a,b*^	77.9	–	–	–
	*Spider3*^*a,b*^	83.9	–	0.575	0.008
	*RaptorX*^*a,b*^	82.2	71.6	0.567	0.027
	*Jpred4*^*a,b*^	76.7	–	–	–
	*DeepSeqVec*	79.1 ± 0.8	67.6 ± 1.0	0.591 ± 0.028	0.012 ± 0.001
	*DeepProf*^*b*^	81.1 ± 0.6	68.3 ± 0.9	0.516 ± 0.028	0.012 ± 0.002
	*DeepProf + SeqVec*^*b*^	82.4 ± 0.7	70.3 ± 1.0	0.585 ± 0.029	0.013 ± 0.003
	*DeepProtVec*	66.0 ± 1.0	54.4 ± 1.3	0.470 ± 0.028	0.011 ± 0.002
	*DeepOneHot*	70.1 ± 0.8	58.5 ± 1.1	0.476 ± 0.028	0.008 ± 0.001
	*Deep BLOSUM65*	70.3 ± 0.8	58.1 ± 1.1	0.488 ± 0.029	0.007 ± 0.001
*CB513*	*NetSurfP-2.0 (hhblits)*^*a,b*^	**85.3**	**72.0**	–	–
	*NetSurfP-1.0*^*a,b*^	78.8	–	–	–
	*Spider3*^*a,b*^	84.5	–	–	–
	*RaptorX*^*a,b*^	82.7	70.6	–	–
	*Jpred4*^*a,b*^	77.9	–	–	–
	*DeepSeqVec*	76.9 ± 0.5	62.5 ± 0.6	–	–
	*DeepProf*^*b*^	80.2 ± 0.4	64.9 ± 0.5	–	–
	*DeepProf + SeqVec*^*b*^	80.7 ± 0.5	66.0 ± 0.5	–	–
	*DeepProtVec*	63.5 ± 0.4	48.9 ± 0.5	–	–
	*DeepOneHot*	67.5 ± 0.4	52.9 ± 0.5	–	–
	*DeepBLOSUM65*	67.4 ± 0.4	53.0 ± 0.5	–	–

Performance comparison for secondary structure (3- vs. 8-classes) and disorder prediction (binary) for the CASP12, TS115 and CB513 data sets. Accuracy (Q3, Q10) is given in percentage. Results marked by ^a^ are taken from NetSurfP-2.0 [46]; the authors did not provide standard errors. Highest numerical values in each column in bold letters. Methods DeepSeqVec, DeepProtVec, DeepOneHot and DeepBLOSUM65 use only information from single protein sequences. Methods using evolutionary information (MSA profiles) are marked by ^b^; these performed best throughout

For the prediction of intrinsic disorder, we observed the same: NetSurfP-2.0 performed best; our implementation of evolutionary information (DeepProf) performed worse (Fig. 1b, Table 1). However, for this task the embeddings alone (DeepSeqVec) performed relatively well, exceeding our in-house implementation of a model using evolutionary information (DeepSeqVec MCC = 0.575–0.591 vs. DeepProf MCC = 0.506–0.516, Table 1). The combination of evolutionary information and embeddings (DeepProf+SeqVec) improved over using evolutionary information alone but did not improve over the *SeqVec* embeddings for disorder. Compared to other methods, the embeddings alone reached similar values (Fig. 1b).

### Per-protein performance close to best

For predicting subcellular localization (cellular compartments) in ten classes, *DeepLoc* [47] is top with Q10 = 78% (Fig. 1c, Table 2). For simplicity, we only tested methods not using evolutionary information/profiles for this task. Our sequence-only embeddings model DeepSeqVec-Loc reached second best performance together with iLoc-Euk [52] at Q10 = 68% (Fig. 1c, Table 2). Unlike the per-residue predictions, for this application the SeqVec embeddings outperformed several popular prediction methods that use evolutionary information by up to 13 percentage points in Q10 (Table 2: DeepSeqVec-Loc vs. methods shown in grayed rows). The gain of the context-dependent SeqVec model introduced here over context-independent versions such as ProtVec (from Word2vec) was even more pronounced than for the per-residue prediction task (Q10 68 ± 1% vs. 42 ± 1%).

**Table 2:** Per-protein predictions: localization and membrane/globular

*Method*	*Localization*		*Membrane/globular*	
	*Q10 (%)*	*Gorodkin (MCC)*	*Q2*	*MCC*
*LocTree2*^*a,b*^	61	0.53		
*MultiLoc2*^*a,b*^	56	0.49		
*CELLO*^*a*^	55	0.45		
*WoLF PSORT*^*a*^	57	0.48		
*YLoc*^*a*^	61	0.53		
*SherLoc2*^*a,b*^	58	0.51		
*iLoc-Euk*^*a,b*^	68	0.64		
*DeepLoc*^*a,b*^	**78**	**0.73**	**92.3**	**0.844**
*DeepSeqVec-Loc*	68 ± 1	0.61 ± 0.01	86.8 ± 1.0	0.725 ± 0.021
*DeepProtVec-Loc*	42 ± 1	0.19 ± 0.01	77.6 ± 1.3	0.531 ± 0.026

Performance for per-protein prediction of subcellular localization and classifying proteins into membrane-bound and water-soluble. Results marked by ^a^ taken from DeepLoc [47]; the authors provided no standard errors. The results reported for *SeqVec* and *ProtVec* were based on single protein sequences, i.e. methods NOT using evolutionary information (neither during training nor testing). All methods using evolutionary information are marked by ^b^; best in each set marked by bold numbers

Performance for the classification into membrane-bound and water-soluble proteins followed a similar trend (Fig. 1d, Table 2): while DeepLoc still performed best (Q2 = 92.3, MCC = 0.844), DeepSeqVec-Loc reached just a few percentage points lower (Q2 = 86.8 ± 1.0, MCC = 0.725 ± 0.021; full confusion matrix Additional file 1: Figure S2). In contrast to this, ProtVec, another method using only single sequences, performed substantially worse (Q2 = 77.6 ± 1.3, MCC = 0.531 ± 0.026).

### Visualizing results

**Fig. 2:**
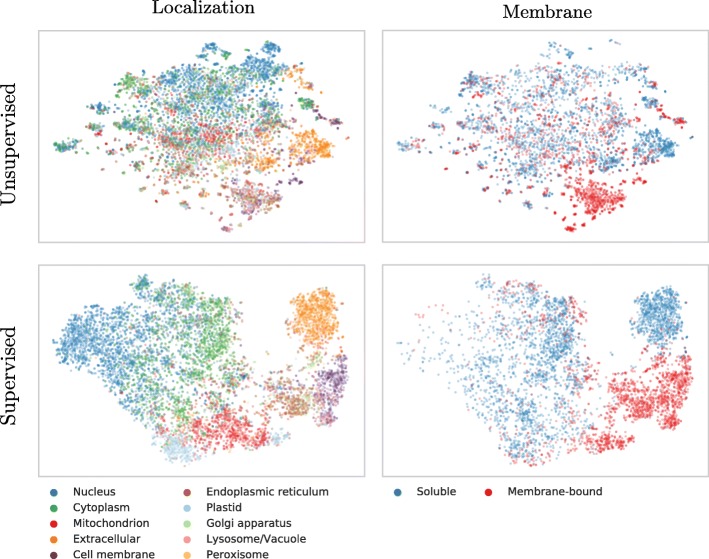
t-SNE representations of SeqVec. Shown are t-SNE projections from embedded space onto a 2D representation; upper row: unsupervised 1024-dimensional “raw” ELMo-based SeqVec embeddings, averaged over all residues in a protein; lower row: supervised 32-dimensional ELMo-based SeqVec embeddings, reduced via per-protein machine learning predictions (data: redundancy reduced set from DeepLoc). Proteins were colored according to their localization (left column) or whether they are membrane-bound or water-soluble (right column). Left and right panel would be identical except for the color, however, on the right we had to leave out some points due to lacking membrane/non-membrane annotations. The upper row suggests that *SeqVec* embeddings capture aspects of proteins without ever seeing labels of localization or membrane, i.e. without supervised training. After supervised training (lower row), this information is transferred to, and further distilled by networks with simple architectures. After training, the power of SeqVeq embeddings to distinguish aspects of function and structure become even more pronounced, sometimes drastically so, as suggested by the almost fully separable clusters in the lower right panel

Lack of insight often triggers the misunderstanding that machine learning methods are black box solutions barring understanding. In order to interpret the *SeqVec* embeddings, we have projected the protein-embeddings of the per-protein prediction data upon two dimensions using t-SNE [53]. We performed this analysis once for the raw embeddings (SeqVec, Fig. 2 upper row) and once for the hidden layer representation of the per-protein network (DeepSeqVec-Loc) after training (Fig. 2 lower row). All t-SNE representations in Fig. 2 were created using 3000 iterations and the cosine distance as metric. The two analyses differed only in that the perplexity was set to 20 for one (*SeqVec*) and 15 for the other (DeepSeqVec-Loc). The t-SNE representations were colored either according to their localization within the cell (left column of Fig. 2) or according to whether they are membrane-bound or water-soluble (right column).

Despite never provided during training, the raw embeddings appeared to capture some signal for classifying proteins by localization (Fig. 2, upper row, left column). The most consistent signal was visible for extra-cellular proteins. Proteins attached to the cell membrane or located in the endoplasmic reticulum also formed well-defined clusters. In contrast, the raw embeddings neither captured a consistent signal for nuclear nor for mitochondrial proteins. Through training, the network improved the signal to reliably classify mitochondrial and plastid proteins. However, proteins in the nucleus and cell membrane continued to be poorly distinguished via t-SNE.

Coloring the t-SNE representations for membrane-bound or water-soluble proteins (Fig. 2, right column), revealed that the raw embeddings already provided well-defined clusters although never trained on membrane prediction (Fig. 2, upper row). After training, the classification was even better (Fig. 2, lower row).

**Fig. 3:**
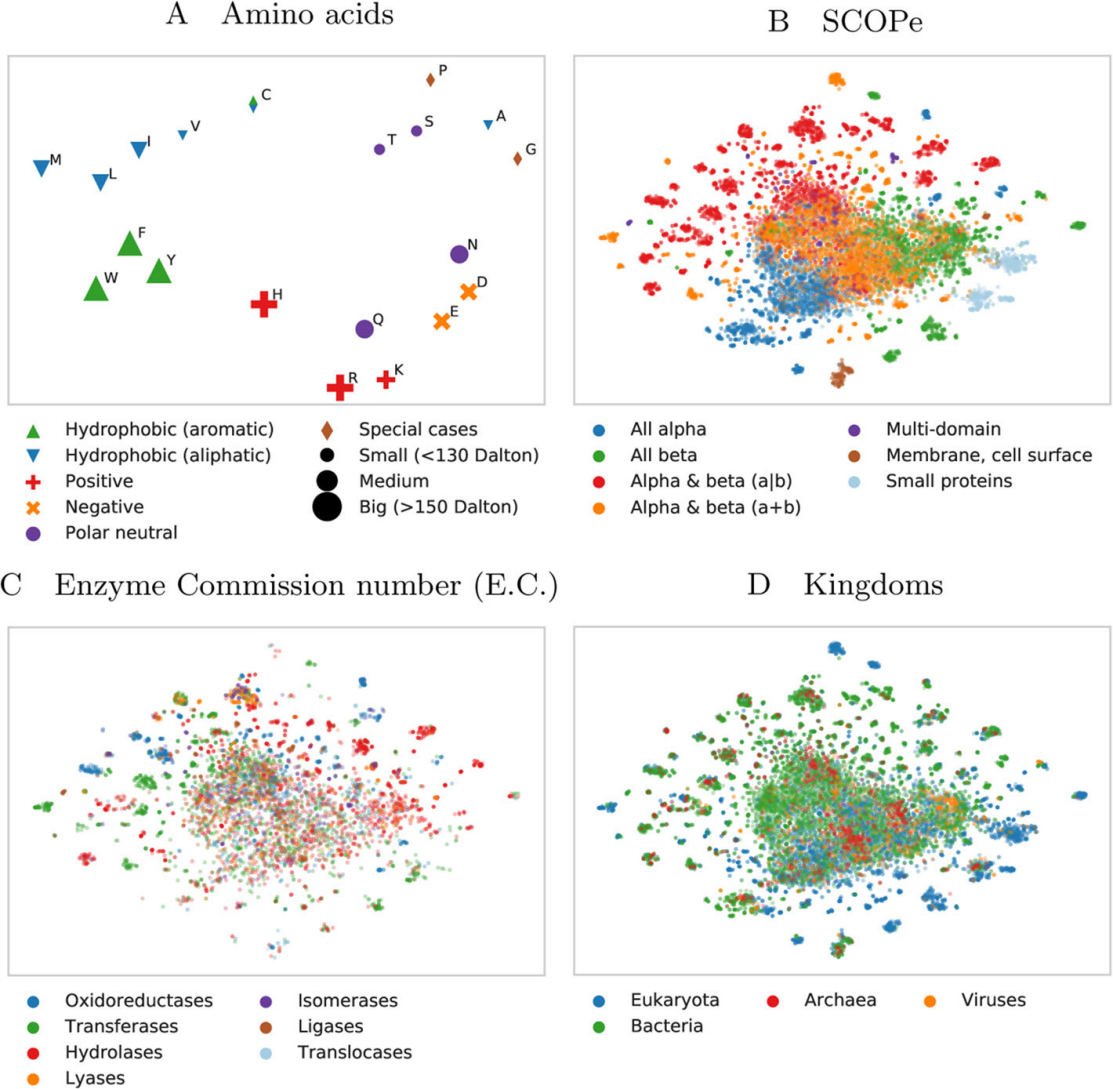
Modeling aspects of the language of life. 2D t-SNE projections of unsupervised *SeqVec* embeddings highlight different realities of proteins and their constituent parts, amino acids. Panels **B** to **D** are based on the same data set (Structural Classification of Proteins – extended (SCOPe) 2.07, redundancy reduced at 40%). For these plots, only subsets of SCOPe containing proteins with the annotation of interest (enzymatic activity C and kingdom D) may be displayed. Panel **A**: the embedding space confirms: the 20 standard amino acids are clustered according to their biochemical and biophysical properties, i.e. hydrophobicity, charge or size. The unique role of Cysteine (C, mostly hydrophobic and polar) is conserved. Panel B: SeqVec embeddings capture structural information as annotated in the main classes in SCOPe without ever having been explicitly trained on structural features. Panel **C**: many small, local clusters share function as given by the main classes in the Enzyme Commission Number (E.C.). Panel **D**: similarly, small, local clusters represent different kingdoms of life

Analogously, we used t-SNE projections to analyze SeqVec embeddings on different levels of complexity inherent to proteins (Fig. 3), ranging from the building blocks (amino acids, Fig. 3a), to secondary structure defined protein classes (Fig. 3b), over functional features (Fig. 3c), and onto the macroscopic level of the kingdoms of life and viruses (Fig. 3d; classifications in panels 3b-3d based on SCOPe [54]). Similar to the results described in [51], our projection of the embedding space confirmed that the model successfully captured bio-chemical and bio-physical properties on the most fine-grained level, i.e. the 20 standard amino acids (Fig. 3a). For example, aromatic amino acids (W, F, Y) are well separated from aliphatic amino acids (A, I, L, M, V) and small amino acids (A, C, G, P, S, T) are well separated from large ones (F, H, R, W, Y). The projection of the letter indicating an unknown amino acid (X), clustered closest to the amino acids alanine (A) and glycine (G) (data not shown). Possible explanations for this could be that the two amino acids with the smallest side chains might be least biased towards other biochemical features like charge and that they are the 2nd (A) and 4th (G) most frequent amino acids in our training set (Additional file 1: Table S1). Rare (O, U) and ambiguous amino acids (Z, B) were removed from the projection as their clustering showed that the model could not learn reasonable embeddings from the very small number of samples.

High-level structural classes as defined in SCOPe (Fig. 3b) were also captured by SeqVec embeddings. Although the embeddings were only trained to predict the next amino acid in a protein sequence, well separated clusters emerged from those embeddings in structure space. Especially, membrane proteins and small proteins formed distinct clusters (note: protein length is not explicitly encoded in *SeqVec*). Also, these results indicated that the embeddings captured complex relationships between proteins which are not directly observable from sequence similarity alone as SCOPe was redundancy reduced at 40% sequence identity. Therefore, the new embeddings could complement sequence-based structural classification as it was shown that the sequence similarity does not necessarily lead to structural similarity [55].

To further investigate the clusters emerging from the SCOPe data set, we colored the same data set based on protein functions (Fig. 3c) and kingdoms (Fig. 3d). This analysis revealed that many of the small, distinct clusters emerged based on protein functions. For instance, transferases and hydrolases formed many small clusters. When increasing the level of abstraction by coloring the proteins according to their kingdoms, we observed certain clusters to be dominated by e.g. eukaryotes. Comparing the different views captured in panels 3B-3D revealed connections, e.g. that all-beta or small proteins dominate in eukaryotes (compare blue and orange islands in Fig. 3b with the same islands in Fig. 3d – colored blue to mark eukaryotes).

### CPU/GPU time used

Due to the sequential nature of LSTMs, the time required to embed a protein grows linearly with protein length. Depending on the available main memory or GPU memory, this process could be massively parallelized. To optimally use available memory, batches are typically based on tokens rather than on sentences. In order to retrieve embeddings, we sorted proteins according to their length and created batches of ≤15 K tokens that could still be handled by a single Nvidia GeForce GTX1080 with 8GB VRAM. The processing of a single protein took on average 0.027 s when applying this batch-strategy to the NetSurfP-2.0 data set (average protein length: 256 residues, i.e. shorter than proteins for which 3D structure is not known). The batch with the shortest proteins (on average 38 residues, corresponding to 15% of the average protein length in the whole data set) required about one tenth (0.003 s per protein, i.e. 11% of that for whole set). The batch containing the longest protein sequences in this data set (1578 residues on average, corresponding to 610% of average protein length in the whole data set), took about six times more (1.5 s per protein, i.e. 556% of that for whole set). When creating SeqVec for the DeepLoc set (average length: 558 residues; as this set does not require a 3D structure, it provides a more realistic view on the distribution of protein lengths), the average processing time for a single protein was 0.08 with a minimum of 0.006 for the batch containing the shortest sequences (67 residues on average) and a maximum of 14.5 s (9860 residues on average). On a single Intel i7–6700 CPU with 64GB RAM, processing time increased by roughly 50% to 0.41 s per protein, with a minimum and a maximum computation time of 0.06 and 15.3 s, respectively. Compared to an average processing time of one hour for 1000 proteins when using evolutionary information directly [46], this implied an average speed up of 120-fold on a single GeForce GTX1080 and 9-fold on a single i7–6700 when predicting structural features; the inference time of DeepSeqVec for a single protein is on average 0.0028 s.

## Discussion

### Transfer-learning alone not top

The context-dependent transfer-learning model ELMo [41] applied to proteins sequences (here dubbed *SeqVec*) clearly succeeded to model the language of protein sequences much better than simple schema (e.g. one-hot encoding), more advanced context-independent language models such as ProtVec (based on Word2vec [42, 43]), more advanced distillations of text-book knowledge (biophysical features used as input for prediction [2, 3]), and also some family-independent information about evolution as represented by the expertise condensed in the BLOSSUM62 matrix. In this sense, our approach worked. However, none of our SeqVec implementations reached today’s best methods: NetSurfP-2.0 for secondary structure and protein disorder and DeepLoc for localization and membrane protein classification (Fig. 1, Table 1, Table 2). Clearly, “just” using SeqVec embeddings to train subsequent prediction methods did not suffice to crack the challenges. Due to computational limitations, testing models trained on larger sequence database, which may over-come this limitation, could not be tested. What about more advanced transfer-learning models, e.g. TransformerXL [56], or different pre-training objectives which model bidirectional contexts, e.g. Bert [57] or XLNet [58]? We have some evidence that transformer-based models might reach further (Elnaggar et al. in preparation), with competing groups already showing promising results [51]. Nevertheless, there is one major reality to remember: we model single protein sequences. Such models might learn the rules for “writing protein sequences” and still miss the constraints imposed by the “survival of the fittest”, i.e. by evolutionary selection.

On the other hand, some of our solutions appeared surprisingly competitive given the simplicity of the architectures. In particular, for the per-protein predictions, for which *SeqVec* clearly outperformed the previously popular *ProtVec* [42] approach and even commonly used expert solutions (Fig. 1, Table 2: no method tested other than the top-of-the-line *DeepLoc* reached higher numerical values). For that comparison, we used the same data sets but could not rigorously compare standard errors (SE) that were unavailable for other methods. Estimating standard errors for our methods suggested the differences to be statistically significant: > 7 SE throughout (exception: DeepLoc (Q10 = 78) and iLoc-Euk(Q10 = 68)). The results for localization prediction implied that frequently used methods using evolutionary information (all marked with shaded boxes in Table 2) did not clearly outperform our simple ELMo-based tool (DeepSeqVec-Loc in Table 2). This was very different for the per-residue prediction tasks: here almost all top methods using evolutionary information numerically outperformed the simple model built on the ELMo embeddings (DeepSeqVec in Fig. 1 and Table 1). However, all models introduced in this work were deliberately designed to be relatively simple to demonstrate the predictive power of *SeqVec*. More sophisticated architectures building up on *SeqVec* embeddings will likely outperform the approaches introduced here.

Combining SeqVec with evolutionary information for per-residue predictions still did not reach the top (set TS115: Q3(NetSurfP-2.0) = 85.3% vs. Q3(DeepProf + SeqVec) = 82.4%, Table 1). This might suggest some limit for the usefulness of the ELMo-based SeqVec embeddings. However, it might also point to the more advanced solutions realized by NetSurfP-2.0 which applies two LSTMs of similar complexity as our entire system (including ELMo) on top of their last step leading to 35 M (35 million) free parameters compared to about 244 K for DeepProf + SeqVec. Twenty times more free parameters might explain some fraction of the success. Due to limited GPU resources, we could not test how much.

Why did the ELMo-based approach improve more (relative to competition) for per-protein than for per-residue predictions? We can only speculate because none of the possible explanations have held consistently for all methods to which we have been applying ELMo embeddings over the recent six months (data not shown). For instance, the per-protein data sets were over two orders of magnitude smaller than those for per-residue predictions; simply because every protein constitutes one sample in the first and protein length samples for the second. SeqVec might have helped more for the smaller data sets because the unlabeled data is pre-processed so meaningful that less information needs to be learned by the ANN during per-protein prediction. This view was strongly supported by the t-SNE [53] results (Fig. 2, Fig. 3): ELMo apparently had learned the “grammar” of the language of life well enough to realize a very rough clustering of structural classes, protein function, localization and membrane/not. Another, yet complementary, explanation for this trend could be that the training of ELMo inherently provides a natural way of summarizing information of proteins of varying length. Other approaches usually learn this summarization step together with the actual prediction tasks which gets increasingly difficult the smaller the data set.

We picked four tasks as proof-of-principle for our ELMo/SeqVec approach. These tasks were picked because recent breakthroughs had been reported (e.g. NetSurfP-2.0 [46] and DeepLoc [47]) and those had made data for training and testing publicly available. We cannot imagine why our findings should not hold true for other tasks of protein prediction and invite the community to apply the *SeqVec* embeddings for their tasks. We assume the SeqVec embeddings to be more beneficial for small than for large data sets. For instance, we expect little or no gain in predicting inter-residue contacts, and more in predicting protein binding sites.

### Good and fast predictions without using evolutionary information

Although our SeqVec embeddings were over five percentage points worse than the best method NetSurfP-2.0 (Table 1: TS115 Q3: 85.3 vs. 79.1), for some proteins (12% in CB513) DeepSeqVec performed better (Additional file 1: Figure S4). We expect those to be proteins with small or incorrect alignments, however, due to the fact that we did not have the alignments available used by NetSurfP-2.0, we could not quite establish the validity of this assumption (analyzing pre-computed alignments from ProteinNet [59] revealed no clear relation of the type: more evolutionary information leads to better prediction). However, the real strength of our solutions is its speed: SeqVec predicted secondary structure and protein disorder over 100-times faster (on a single 8GB GPU) than NetSurfP-2.0 when counting the time it needs to retrieve the evolutionary information summarized in alignment profiles although using the fastest available alignment method, namely MMseqs2 [36] which already can reach speed-up values of 100-times over PSI-BLAST [33]. For those who do not have enough resources for running MMSeqs2 and therefore have to rely on PSI-BLAST, the speed-up of our prediction becomes 10,000-fold. Even the 100-fold speed-up is so substantial that for some applications, the speedup might outweigh the reduction in performance. Embedding based approaches such as *SeqVec* suggest a promising solution toward solving one of the biggest challenges for computational biology: how to efficiently handle the exponentially increasing number of sequences in protein databases? Here, we showed that relevant information from large unannotated biological databases can be compressed into embeddings that condense and abstract the underlying biophysical principles. These embeddings, essentially the weights of a neural network, help as input to many problems for which smaller sets of annotated data are available (secondary structure, disorder, localization). Although the compression step needed to build the *SeqVec* model is very GPU-intensive, it can be performed in a centralized way using large clusters. After training, the model can be shipped and used on any consumer hardware. Such solutions are ideal to support researches without access to expensive cluster infrastructure.

### Modeling the language of life?

SeqVec, our pre-trained ELMo adaption, learned to model a probability distribution over a protein sequence. The sum over this probability distribution constituted a very informative input vector for any machine learning task trying to predict protein features. It also picked up context-dependent protein motifs without explicitly explaining what these motifs are relevant for. In contrast, context-independent tools such as *ProtVec* [42] will always create the same vectors regardless of the residues surrounding this k-mer in a protein sequence.

Our hypothesis had been that the ELMo-based *SeqVec* embeddings trained on large databases of un-annotated protein sequences could extract a *probabilistic model of the language of life* in the sense that the resulting system will extract aspects relevant both for per-residue and per-protein prediction tasks. All results presented here have added independent evidence in full support of this hypothesis. For instance, the three state per-residue accuracy for secondary structure prediction improved by over eight percentage points through ELMo (Table 1, e.g. Q3: 79.1 vs. 70.3%), the per-residue MCC for protein disorder prediction also increased substantially (Table 1, e.g. MCC: 0.591 vs. 0.488). On the per-protein level, the improvement over the previously popular tool extracting “meaning” from proteins, *ProtVec*, was even more substantial (Table 1: e.g. Q10: 68% vs. 42%). We could demonstrate this reality even more directly using the t-SNE [53] results (Fig. 2 and Fig. 3): different levels of complexity ranging from single amino acids, over some localizations, structural features, functions and the classification of membrane/non-membrane had been implicitly learned by *SeqVec* without training. Clearly, our ELMo-driven implementation of transfer-learning fully succeeded to model some aspects of the language of life as proxied by protein sequences. How much more will be possible? Time will tell.

## Conclusion

We have shown that it is possible to capture and transfer knowledge, e.g. biochemical or biophysical properties, from a large unlabeled data set of protein sequences to smaller, labelled data sets. In this first proof-of-principle, our comparably simple models have already reached promising performance for a variety of per-residue and per-protein prediction tasks obtainable from only single protein sequences as input, that is: without any direct evolutionary information, i.e. without profiles from multiple sequence alignments of protein families. This reduces the dependence on the time-consuming and computationally intensive calculation of protein profiles, allowing the prediction of per-residue and per-protein features of a whole proteome within less than an hour. For instance, on a single GeForce GTX 1080, the creation of embeddings and predictions of secondary structure and subcellular localization for the whole human proteome took about 32 min. Building more sophisticated architectures on top of *SeqVec* might increase sequence-based performance further.

Our new *SeqVec* embeddings may constitute an ideal starting point for many different applications in particular when labelled data are limited. The embeddings combined with evolutionary information might even improve over the best available methods, i.e. enable high-quality predictions. Alternatively, they might ease high-throughput predictions of whole proteomes when used as the only input feature. Alignment-free predictions bring speed and improvements for proteins for which alignments are not readily available or limited, such as for intrinsically disordered proteins, for the Dark Proteome, or for particular unique inventions of evolution. The trick was to tap into the potential of Deep Learning through transfer learning from large repositories of unlabeled data by modeling the language of life.

## Methods

### Data

UniRef50 training of *SeqVec:* We trained ELMo on UniRef50 [32], a sequence redundancy-reduced subset of the UniProt database clustered at 50% pairwise sequence identity (PIDE). It contained 25 different letters (20 standard and 2 rare amino acids (U and O) plus 3 special cases describing either ambiguous (B, Z) or unknown amino acids (X); Additional file 1: Table S1) from 33 M proteins with 9,577,889,953 residues. In order to train ELMo, each protein was treated as a sentence and each amino acid was interpreted as a single word.

Visualization of embedding space: The current release of the “Structural Classification Of Proteins” (SCOPe, [54]) database (2.07) contains 14,323 proteins at a redundancy level of 40%. Functions encoded by the Enzyme Commission number (E.C., [60]) were retrieved via the “Structure Integration with Function, Taxonomy and Sequence” (SIFTS) mapping [61]. SIFTS allows, among other things, a residue-level mapping between UniProt and PDB entries and a mapping from PDB identifiers to E.C.s. If no function annotation was available for a protein or if the same PDB identifier was assigned to multiple E.C.s, it was removed from Fig. 3c. Taxonomic identifiers from UniProt were used to map proteins to one of the 3 kingdoms of life or to viruses. Again, proteins were removed if no such information was available. The number of iterations for the t-SNE projections was set again to 3000 and the perplexity was adjusted (perplexity = 5 for Fig. 3a and perplexity = 30 for Fig. 3b-d).

Per-residue level: secondary structure & intrinsic disorder (*NetSurfP-2.0*). To simplify comparability, we used the data set published with a recent method seemingly achieving the top performance of the day in secondary structure prediction, namely *NetSurfP-2.0* [46]. Performance values for the same data set exist also for other recent methods such as *Spider3* [62], *RaptorX* [63, 64] and *JPred4* [65]. The set contains 10,837 sequence-unique (at 25% PIDE) proteins of experimentally known 3D structures from the PDB [66] with a resolution of 2.5 Å (0.25 nm) or better, collected by the PISCES server [67]. DSSP [68] assigned secondary structure and intrinsically disordered residues are flagged (residues without atomic coordinates, i.e. REMARK-465 in the PDB file). The original seven DSSP states (+ 1 for unknown) were mapped upon three states using the common convention: [G,H,I] → H (helix), [B,E] → E (strand), all others to O (other; often misleadingly referred to as *coil* or *loop*). As the authors of NetSurfP-2.0 did not include the raw protein sequences in their public data set, we used the SIFTS file to obtain the original sequence. Only proteins with identical length in SIFTS and NetSurfP-2.0 were used. This filtering step removed 56 sequences from the training set and three from the test sets (see below: two from CB513, one from CASP12 and none from TS115). We randomly selected 536 (~ 5%) proteins for early stopping (*cross-training*), leaving 10,256 proteins for training. All published values referred to the following three test sets (also referred to as validation set): **TS115** [69]: 115 proteins from high-quality structures (< 3 Å) released after 2015 (and at most 30% PIDE to any protein of known structure in the PDB at the time); **CB513** [70]: 513 non-redundant sequences compiled 20 years ago (511 after SIFTS mapping); **CASP12** [71]: 21 proteins taken from the CASP12 free-modelling targets (20 after SIFTS mapping; all 21 fulfilled a stricter criterion toward non-redundancy than the two other sets; non-redundant with respect to all 3D structures known until May 2018 and all their relatives). Each of these sets covers different aspects of the secondary structure prediction problem: CB513 and TS115 only use structures determined by X-ray crystallography and apply similar cutoffs with respect to redundancy (30%) and resolution (2.5–3.0 Å). While these serve as a good proxy for a baseline performance, CASP12 might better reflect the true generalization capability for unseen proteins as it includes structures determined via NMR and Cryo-EM. Also, the strict redundancy reduction based on publication date reduces the bias towards well studied families. Nevertheless, toward our objective of establishing a proof-of-principle, these sets sufficed. All test sets had fewer than 25% PIDE to any protein used for training and cross-training (ascertained by the *NetSurfP-2.0* authors). To compare methods using evolutionary information and those using our new word embeddings, we took the *HHblits* profiles published along with the NetSurfP-2.0 data set.

Per-protein level: subcellular localization & membrane proteins (DeepLoc). Subcellular localization prediction was trained and evaluated using the *DeepLoc* data set [47] for which performance was measured for several methods, namely: LocTree2 [72], MultiLoc2 [73], SherLoc2 [74], CELLO [75], iLoc-Euk [52], WoLF PSORT [76] and YLoc [77]. The data set contained proteins from UniProtKB/Swiss-Prot [78] (release: 2016_04) with experimental annotation (code: ECO:0000269). The *DeepLoc* authors mapped these annotations to ten classes, removing all proteins with multiple annotations. All these proteins were also classified into *water-soluble* or *membrane-bound* (or as *unknown* if the annotation was ambiguous). The resulting 13,858 proteins were clustered through PSI-CD-HIT [79, 80] (version 4.0; at 30% PIDE or Eval< 10^− 6^). Adding the requirement that the alignment had to cover 80% of the shorter protein, yielded 8464 clusters. This set was split into training and testing by using the same proteins for testing as the authors of DeepLoc. The training set was randomly sub-divided into 90% for training and 10% for determining early stopping (cross-training set).

### Embedding terminology and related work

**Fig. 4:**
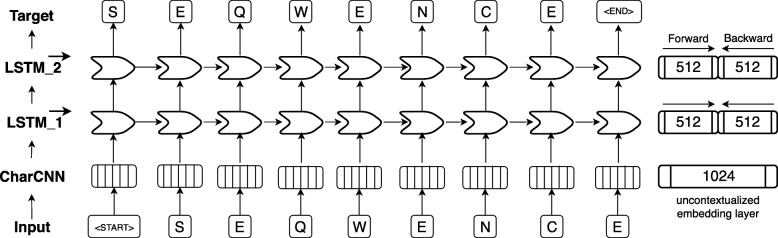
ELMo-based architecture adopted for SeqVec. First, an input sequence, e.g. “S E Q W E N C E” (shown at bottom row), is padded with special tokens indicating the start (“<start>”) and the end (“<end>”) of the sentence (here: protein sequences). On the 2nd level (2nd row from bottom), character convolutions (CharCNN, [94]) map each word (here: amino acid) onto a fixed-length latent space (here: 1024-dimensional) without considering information from neighboring words. On the third level (3rd row from bottom), the output of the CharCNN-layer is used as input by a bidirectional Long Short Term Memory (LSTM, [45]) which introduces context-specific information by processing the sentence (protein sequence) sequentially. For simplicity, only the forward pass of the bi-directional LSTM-layer is shown (here: 512-dimensional). On the fourth level (4th row from bottom), the second LSTM-layer operates directly on the output of the first LSTM-layer and tries to predict the next word given all previous words in a sentence. The forward and backward pass are optimized independently during training in order to avoid information leakage between the two directions. During inference, the hidden states of the forward and backward pass of each LSTM-layer are concatenated to a 1024-dimensional embedding vector summarizing information from the left and the right context

One-hot encoding (also known as *sparse encoding*) assigns each word (referred to as token in NLP) in the vocabulary an integer N used as the Nth component of a vector with the dimension of the vocabulary size (number of different words). Each component is binary, i.e. either 0 if the word is not present in a sentence/text or 1 if it is. This encoding drove the first application of machine learning that clearly improved over all other methods in protein prediction [1–3]. TF-IDF represents tokens as the product of “frequency of token in data set” times “inverse frequency of token in document”. Thereby, rare tokens become more relevant than common words such as “the” (so called *stop words*). This concept resembles that of using k-mers for database searches [33], clustering [81], motifs [82, 83], and prediction methods [72, 76, 84–88]. Context-insensitive word embeddings replaced expert features, such as TF-IDF, by algorithms that extracted such knowledge automatically from unlabeled corpus such as Wikipedia, by either predicting the neighboring words, given the center word (skip-gram) or vice versa (CBOW). This became known in *Word2Vec* [43] and showcased for computational biology through *ProtVec* [43, 89]. ProtVec assumes that every token or word consists of three consecutive residues (amino acid 3-mers). During training, each protein sequence in *SwissProt* [78] is split into overlapping 3-mers and the skip-gram version of *word2vec* is used to predict adjacent 3-mers, given the 3-mer at the center. After training, protein sequences can be split into overlapping 3-mers which are mapped onto a 100-dimensional latent space. More specialized implementations are *mut2vec* [90] learning mutations in cancer, and *phoscontext2vec* [91] identifying phosphorylation sites. Even though the performance of context-insensitive approaches was pushed to its limits by adding sub-word information (FastText [92]) or global statistics on word co-occurance (GloVe [93]), their expressiveness remained limited because the models inherently assigned the same vector to the same word, regardless of its context. Context-sensitive word embeddings started a new wave of word embedding techniques for NLP in 2018: the embedding renders the meaning of words and phrases such as “*paper tiger”* dependent upon the context, allowing to account for the ambiguous meanings of words. Popular examples like ELMo [41] and Bert [57] have achieved state-of-the-art results in several NLP tasks. Both require substantial GPU computing power and time to be trained from scratch. One of the main differences between ELMo and Bert is their pre-training objective: while auto-regressive models like ELMo predict the next word in a sentence given all previous words, autoencoder-based models like Bert predict masked-out words given all words which were not masked out. However, in this work we focused on ELMo as it allows processing of sequences of variable length. The original ELMo model consists of a single, context-insensitive CharCNN [94] over the characters in a word and two layers of bidirectional LSTMs that introduce the context information of surrounding words (Fig. 4). The CharCNN transforms all characters within a single word via an embedding layer into vector space and runs multiple CNNs of varying window size (here: ranging from 1 to 7) and number of filters (here: 32, 64, …, 1024). In order to obtain a fixed-dimensional vector for each word, regardless of its length, the output of the CNNs is max-pooled and concatenated. This feature is crucial for NLP in order to be able to process words of variable length. As our words consist only of single amino acids, this layer learns an uncontextualized mapping of single amino acids onto a latent space. The first bi-directional LSTM operates directly on the output of the CharCNN, while the second LSTM layer takes the output of the first LSTM as input. Due to their sequential nature, the LSTM layers render the embeddings dependent on their context as their internal state always depends on the previous hidden state. However, the bidirectionality of the LSTMs would lead to information leakage, rendering the training objective trivial, i.e. the backward pass had already seen the word which needs to be predicted in the forward pass. This problem is solved by training the forward and the backward pass of the LSTMs independently, i.e. the forward pass is conditioned only on words to its left and vice versa. During inference the internal states of both directions are concatenated allowing the final embeddings to carry information from both sides of the context. As described in the original ELMo publication, the weights of the forward and the backward model are shared in order to reduce the memory overhead of the model and to combat overfitting. Even though, the risk of overfitting is small due to the high imbalance between number of trainable parameters (93 M) versus number of tokens (9.3B), dropout at a rate of 10% was used to reduce the risk of overfitting. This model is trained to predict the next amino acid given all previous amino acids in a protein sequence. To the best of our knowledge, the context-sensitive ELMo has not been adapted to protein sequences, yet.

### ELMo adaptation

In order to adapt ELMo [41] to protein sequences, we used the standard ELMo configuration with the following changes: (i) reduction to 28 tokens (20 standard and 2 rare (U,O) amino acids + 3 special tokens describing ambiguous (B,Z) or unknown (X) amino acids + 3 special tokens for ELMo indicating padded elements (‘<MASK>’) or the beginning (‘<S>’) or the end of a sequence (‘</S>’)), (ii) increase number of unroll steps to 100 to account for the increased length of protein sequences compared to sentences in natural languages, (iii) decrease number of negative samples to 20, (iv) increase token number to 9,577,889,953. After pre-training the ELMo architecture (1 CharCNN, 2 LSTM-Layers, see “Embedding terminology and related work” section and Fig. 4 for more details) with our parameters on UniRef50, the embedding model takes a protein sequence of arbitrary length and returns 3076 features for each residue in the sequence. These 3076 features were derived by concatenating the outputs of the three layers of ELMo, each describing a token with a vector of length 1024. The LSTM layers were composed of the embedding of the forward pass (first 512 dimensions) and the backward pass (last 512 dimensions). In order to demonstrate the general applicability of ELMo or *SeqVec* and to allow for easy integration into existing models, we neither fine-tuned the pre-trained model on a specific prediction task, nor optimized the combination of the three internal layers. Thus, researchers could just replace (or concatenate) their current machine learning inputs with our embeddings to boost their task-specific performance. Furthermore, it will simplify the development of custom models that fit other use-cases. For simplicity, we summed the components of the three 1024-dimensional vectors to form a single 1024-dimensional feature vector describing each residue in a protein.

### Using SeqVec for predicting protein features

**Fig. 5:**
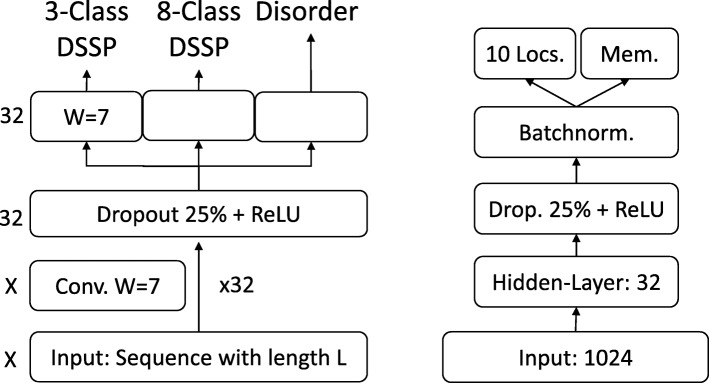
Prediction tasks’ architectures. On the left the architecture of the model used for the per-residue level predictions (secondary structure and disorder) is sketched, on the right that used for per-protein level predictions (localization and membrane/not membrane). The ‘X’, on the left, indicates that different input features corresponded to a difference in the number of input channels, e.g. 1024 for *SeqVec* or 50 for profile-based input. The letter ‘W’ refers to the window size of the corresponding convolutional layer (W = 7 implies a convolution of size 7 × 1)

On the per-residue level, the predictive power of the new *SeqVec* embeddings was demonstrated by training a small two-layer Convolutional Neural Network (CNN) in PyTorch using a specific implementation [95] of the ADAM optimizer [96], cross-entropy loss, a learning rate of 0.001 and a batch size of 128 proteins. The first layer (in analogy to the sequence-to-structure network of earlier solutions [2, 3]) consisted of 32-filters each with a sliding window-size of w = 7. The second layer (structure-to-structure [2, 3]) created the final predictions by applying again a CNN (w = 7) over the output of the first layer. These two layers were connected through a rectified linear unit (ReLU) and a dropout layer [97] with a dropout-rate of 25% (Fig. 5, left panel). This simple architecture was trained independently on six different types of input, resulting in different number of free parameters. (i) DeepProf (14,000 = 14 k free parameters): Each residue was described by a vector of size 50 which included a one-hot encoding (20 features), the profiles of evolutionary information (20 features) from HHblits as published previously [46], the state transition probabilities of the Hidden-Markov-Model (7 features) and 3 features describing the local alignment diversity. (ii) DeepSeqVec (232 k free parameters): Each protein sequence was represented by the output of SeqVec. The resulting embedding described each residue as a 1024-dimensional vector. (iii) DeepProf+SeqVec (244 k free parameters): This model simply concatenated the input vectors used in (i) and (ii). (iv) DeepProtVec (25 k free parameters): Each sequence was split into overlapping 3-mers each represented by a 100-dimensional ProtVec [42]. (v) DeepOneHot (7 k free parameters): The 20 amino acids were encoded as one-hot vectors as described above. Rare amino acids were mapped to vectors with all components set to 0. Consequently, each protein residue was encoded as a 20-dimensional one-hot vector. (vi) DeepBLOSUM65 (8 k free parameters): Each protein residue was encoded by its BLOSUM65 substitution matrix [98]. In addition to the 20 standard amino acids, BLOSUM65 also contains substitution scores for the special cases B, Z (ambiguous) and X (unknown), resulting in a feature vector of length 23 for each residue.

On the per-protein level, a simple feed-forward neural network was used to demonstrate the power of the new embeddings. In order to ensure equal-sized input vectors for all proteins, we averaged over the 1024-dimensional embeddings of all residues in a given protein resulting in a 1024-dimensional vector representing any protein in the data set. ProtVec representations were derived the same way, resulting in a 100-dimensional vector. These vectors (either 100-or 1024 dimensional) were first compressed to 32 features, then dropout with a dropout rate of 25%, batch normalization [99] and a rectified linear Unit (ReLU) were applied before the final prediction (Fig. 5, right panel). In the following, we refer to the models trained on the two different input types as (i) DeepSeqVec-Loc (33 k free parameters): average over SeqVec embedding of a protein as described above and (ii) DeepProtVec-Loc (320 free parameters): average over ProtVec embedding of a protein. We used the following hyper-parameters: learning rate: 0.001, Adam optimizer with cross-entropy loss, batch size: 64. The losses of the individual tasks were summed before backpropagation. Due to the relatively small number of free parameters in our models, the training of all networks completed on a single Nvidia GeForce GTX1080 within a few minutes (11 s for DeepProtVec-Loc, 15 min for DeepSeqVec).

### Evaluation measures

To simplify comparisons, we ported the evaluation measures from the publications we derived our data sets from, i.e. those used to develop *NetSurfP-2.0* [46] and *DeepLoc* [47]. All numbers reported constituted averages over all proteins in the final test sets. This work aimed at a proof-of-principle that the *SeqVec* embedding contain predictive information. In the absence of any claim for state-of-the-art performance, we did not calculate any significance values for the reported values.

Per-residue performance: Toward this end, we used the standard three-state per-residue accuracy (Q3 = percentage correctly predicted in either helix, strand, other [2]) along with its eight-state analog (Q8). Predictions of intrinsic disorder were evaluated through the Matthew’s correlation coefficient (MCC [100]) and the False-Positive Rate (FPR) as those are more informative for tasks with high class imbalance. For completeness, we also provided the entire confusion matrices for both secondary structure prediction problems (Additional file 1: Figure S2). Standard errors were calculated over the distribution of each performance measure for all proteins.

Per-protein performance: The predictions whether a protein was membrane-bound or water-soluble were evaluated by calculating the two-state per set accuracy (Q2: percentage of proteins correctly predicted), and the MCC. A generalized MCC using the Gorodkin measure [101] for K (=10) categories as well as accuracy (Q10), was used to evaluate localization predictions. Standard errors were calculated using 1000 bootstrap samples, each chosen randomly by selecting a sub-set of the predicted test set that had the same size (draw with replacement).

## Supplementary information

**Additional file 1:** Supporting online material (SOM) for: Modeling aspect of the language of life through transfer-learning protein sequences **Figure 1.** ELMo perplexity **Figure 2.** Confusion matrices for per-protein predictions using DeepSeqVec-Loc **Figure 3.** Confusion matrices for secondary structure predictions of DeepSeqVec **Figure 4.** Comparison of secondary structure prediction performance (Q3) between Netsurfp-2.0 and DeepSeqVec **Table S1.** Amino acid occurrences in UniRef50

## Data Availability

The pre-trained ELMo-based SeqVec model and a description on how to implement the embeddings into existing methods can be found here: https://github.com/Rostlab/SeqVec . Accessed 2nd May 2019. Predictions on secondary structure, disorder and subcellular localization based on SeqVec can be accessed under: https://embed.protein.properties . Accessed 2nd May 2019. The NetSurfP-2.0 data set [46] used for the evaluation of SeqVec on the task of secondary structure and disorder prediction are publicly available under: http://www.cbs.dtu.dk/services/NetSurfP/ . Accessed 2nd May 2019. The DeepLoc data set [47] used for the evaluation of SeqVec on the task of subcellular localization prediction are publicly available under: http://www.cbs.dtu.dk/services/DeepLoc/data.php . Accessed 2nd May 2019.

## Abbreviations

1D: One-dimensional – information representable in a string such as secondary structure or solvent accessibility
3D structure: Three-dimensional coordinates of protein structure
3D: Three-dimensional
ELMo: Embeddings from Language Models
MCC: Matthews-Correlation-Coefficient
MSA: Multiple sequence alignment
ProtVec: Context-independent embeddings from Word2vec-type approaches
Q10: Ten-state localization per-protein accuracy
Q3: Three-state secondary structure per-residue accuracy
Q8: Eight-state secondary structure per-residue accuracy
RSA: Relative solvent accessibility
SE: Standard error
SeqVec: embeddings introduced here, extracted by modeling un-annotated UniRef50 protein sequences with ELMo
