# Supplementary material for: Modeling aspects of the language of life through transfer-learning protein sequences
Source: BMC Bioinformatics. 2019 Dec 17;20:723. doi: 10.1186/s12859-019-3220-8 (PMC6918593; doi:10.1186/s12859-019-3220-8)
Supplement: Supplementary file 1 — Additional file 1: Supporting online material (SOM) for: Modeling aspect of the language of life through transfer-learning protein sequences Figure 1. ELMo perplexity Figure 2. Confusion matrices for per-protein predictions using DeepSeqVec-Loc Figure 3. Confusion matrices for secondary structure predictions of DeepSeqVec Figure 4. Comparison of secondary structure prediction performance (Q3) between Netsurfp-2.0 and DeepSeqVec Table S1. Amino acid occurrences in UniRef50 [file 12859_2019_3220_MOESM1_ESM.doc]

Supporting online material (SOM) for:
Modeling aspect of the language of life through transfer-learning protein sequences

Michael Heinzinger, Ahmed Elnaggar, Yu Wang, Christian Dallago, Dmitrii Nechaev, Florian Matthes & Burkhard Rost

# Table of Contents for SOM

[Table of Contents for SOM 1](#__RefHeading___Toc25673561)

[Short description of SOM 1](#__RefHeading___Toc25673562)

[SOM: Modelling the language of life 1](#__RefHeading___Toc25673563)

[Fig. S1: ELMo perplexity 1](#__RefHeading___Toc25673564)

[Fig. S2: Confusion matrices for per-protein predictions using SeqVec 2](#__RefHeading___Toc25673565)

[Fig. S3: Confusion matrices for secondary structure predictions of DeepSeqVec 3](#__RefHeading___Toc25673566)

[Fig. S4: Comparison of secondary structure prediction performance (Q3) between Netsurfp-2.0 and DeepSeqVec 4](#__RefHeading___Toc25673567)

[Table S1: Amino acid occurrences in UniRef50 ^◊^ 5](#__RefHeading___Toc25673568)

# Short description of SOM

The evolution of the model’s uncertainty (or perplexity) when predicting the next token during training is shown in Fig. SOM_1. The vocabulary and the number of occurrences of the tokens used to train ELMo are shown in Table SOM_1.

Confusion matrices for predictions on the level of residues (Figure SOM_2) and on the level of whole proteins (Figure SOM_3) are given in the following. Also, a comparison of secondary structure prediction performance (Q3) between Netsurfp-2.0 and DeepSeqVec is given (Figure SOM_4).

# SOM: Modelling the language of life

###### Fig. S1: ELMo perplexity


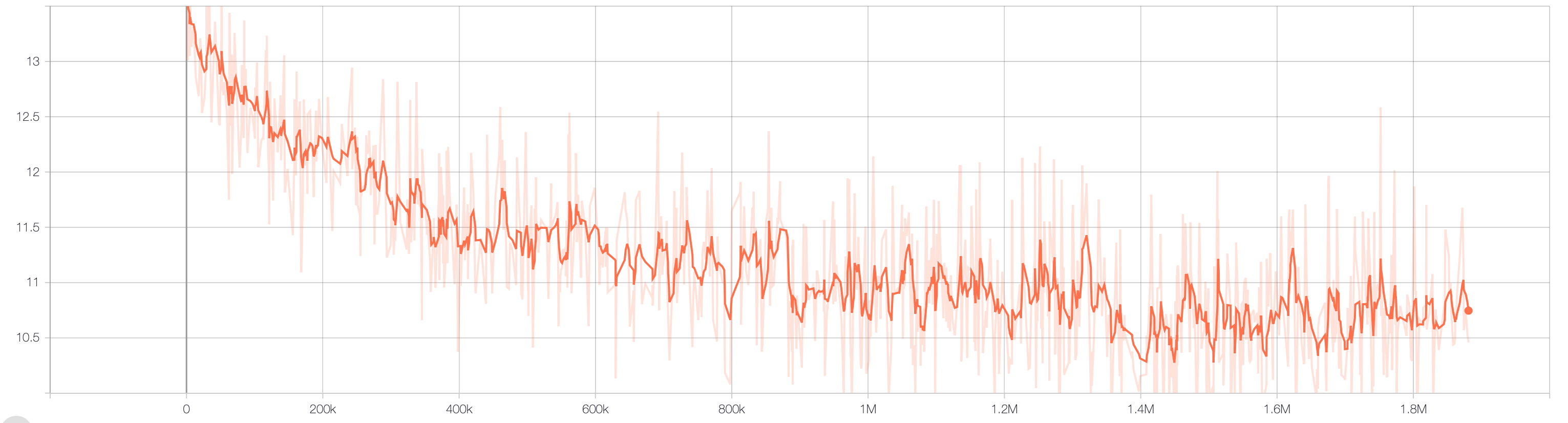


**Fig. S1: ELMo perplexity.** The perplexity defines the uncertainty of a model when predicting the next token (here: amino acid), given all previous tokens in a sequence. Lower values indicate less uncertainty. This measure can be used to monitor the training progress (y-axis: perplexity) over time (x-axis: number of training steps). Here, the learning progress of the proposed ELMo-based SeqVec is shown while being trained on UniRef50.

###### Fig. S2: Confusion matrices for per-protein predictions using SeqVec


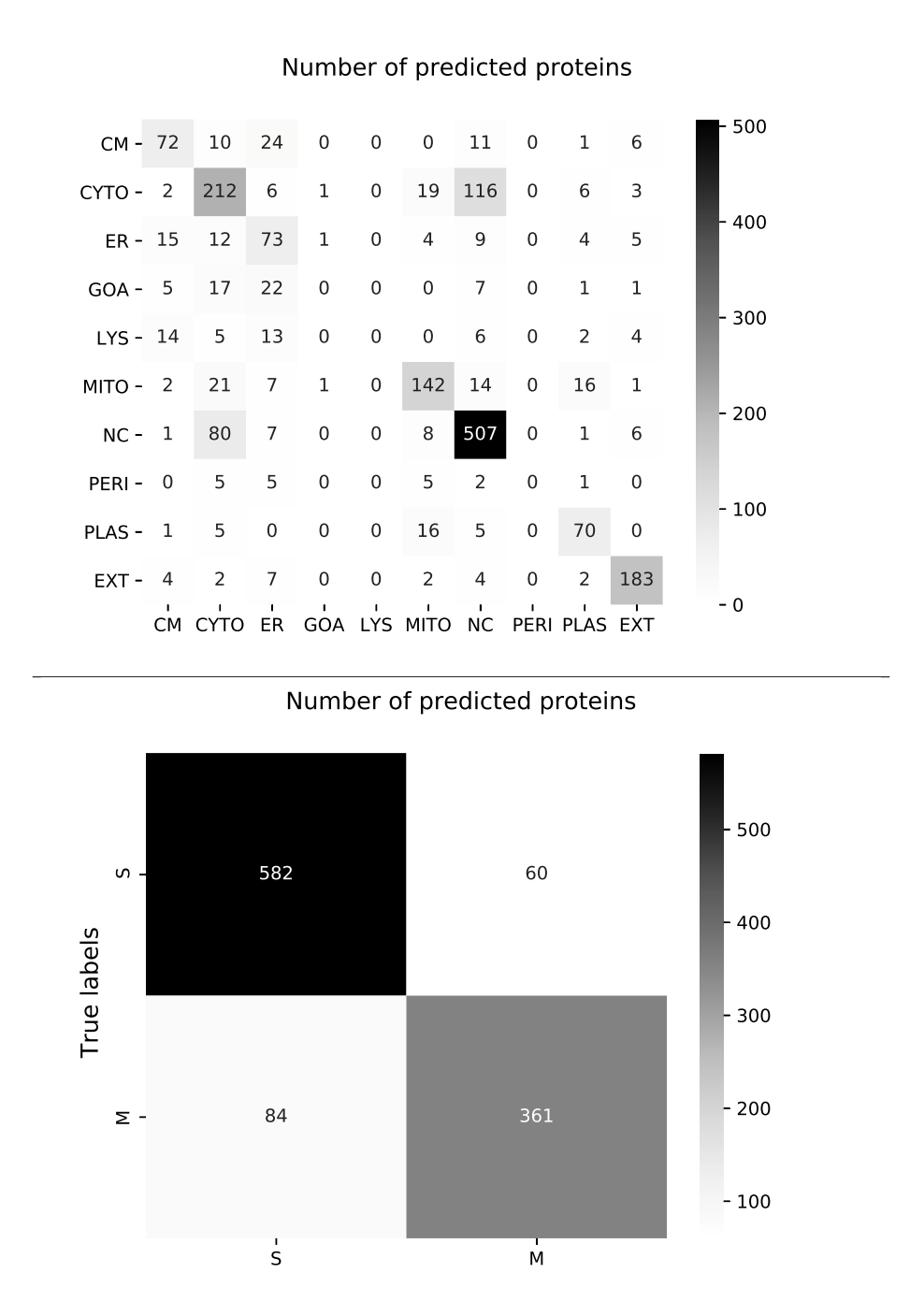


**Fig. S2: Confusion matrices for per-protein predictions using only DeepSeqVec-Loc.** The confusion matrix for localization prediction is shown in the upper row, while results for membrane-bound versus water-soluble are given in the lower row. Again, each confusion matrix summarizes true labels (rows) and predictions (columns). Localizations are abbreviated for simplicity (CM=cell membrane, CYTO=cytoplasm, ER=endoplasmic reticulum, GOA=golgi apparatus, LYS=Lysosome/Vacuole, MITO=mitochondrion, NC=nucleus, PERI=peroxisome, PLAS=plastid, EXT=extracellular).

###### Fig. S3: Confusion matrices for secondary structure predictions of DeepSeqVec


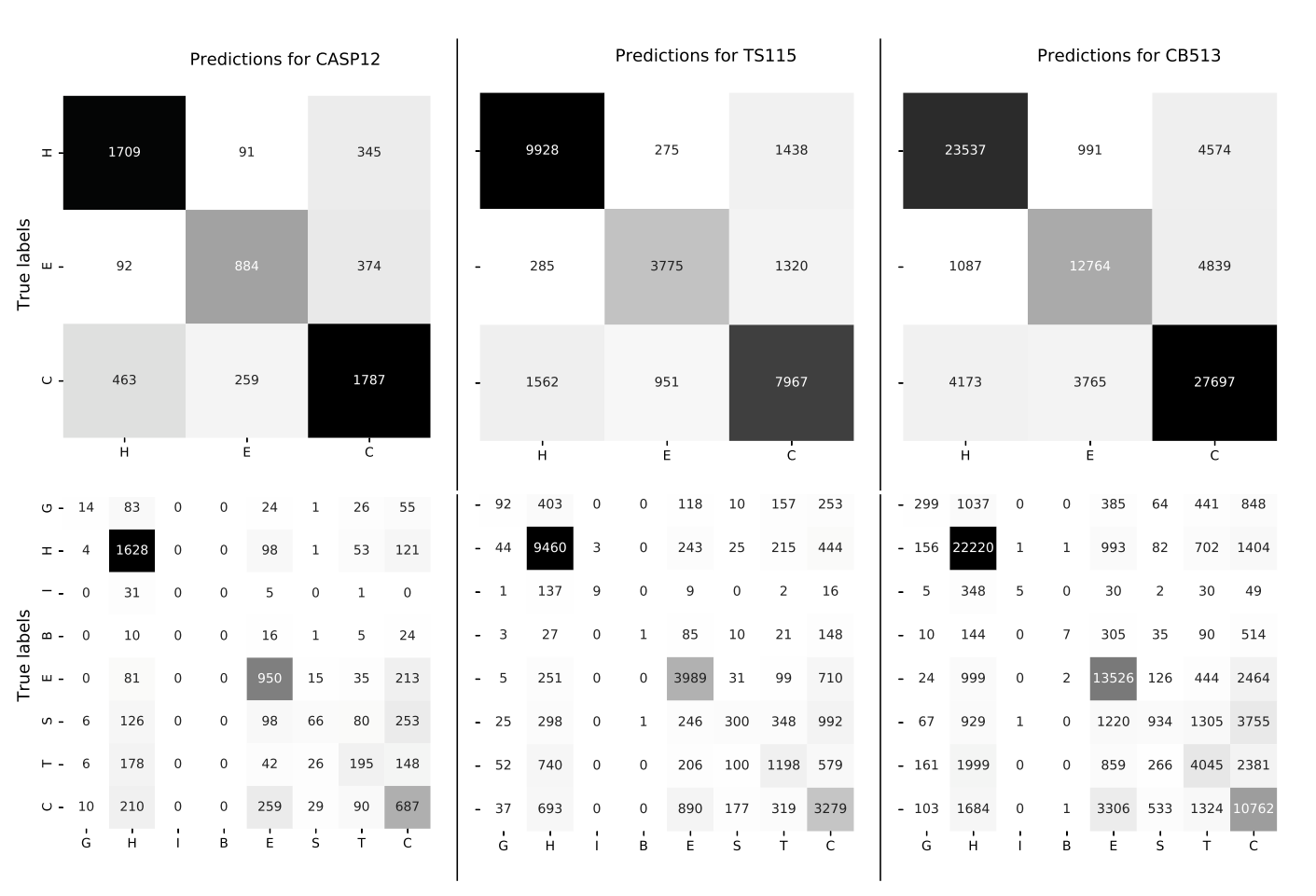


**Fig. S3: Confusion matrices for secondary structure predictions of DeepSeqVec.** The confusion matrices for 3-state secondary structure prediction are given in the upper row, 8-state confusion matrices in the lower row. The columns refer to the three different test sets: CASP12, TS115, CB513. Each matrix summarizes the true labels (rows) and the predictions (columns) for each of the sets or tasks. Numbers on the diagonal reflect correct predictions.

###### Fig. S4: Comparison of secondary structure prediction performance (Q3) between Netsurfp-2.0 and DeepSeqVec


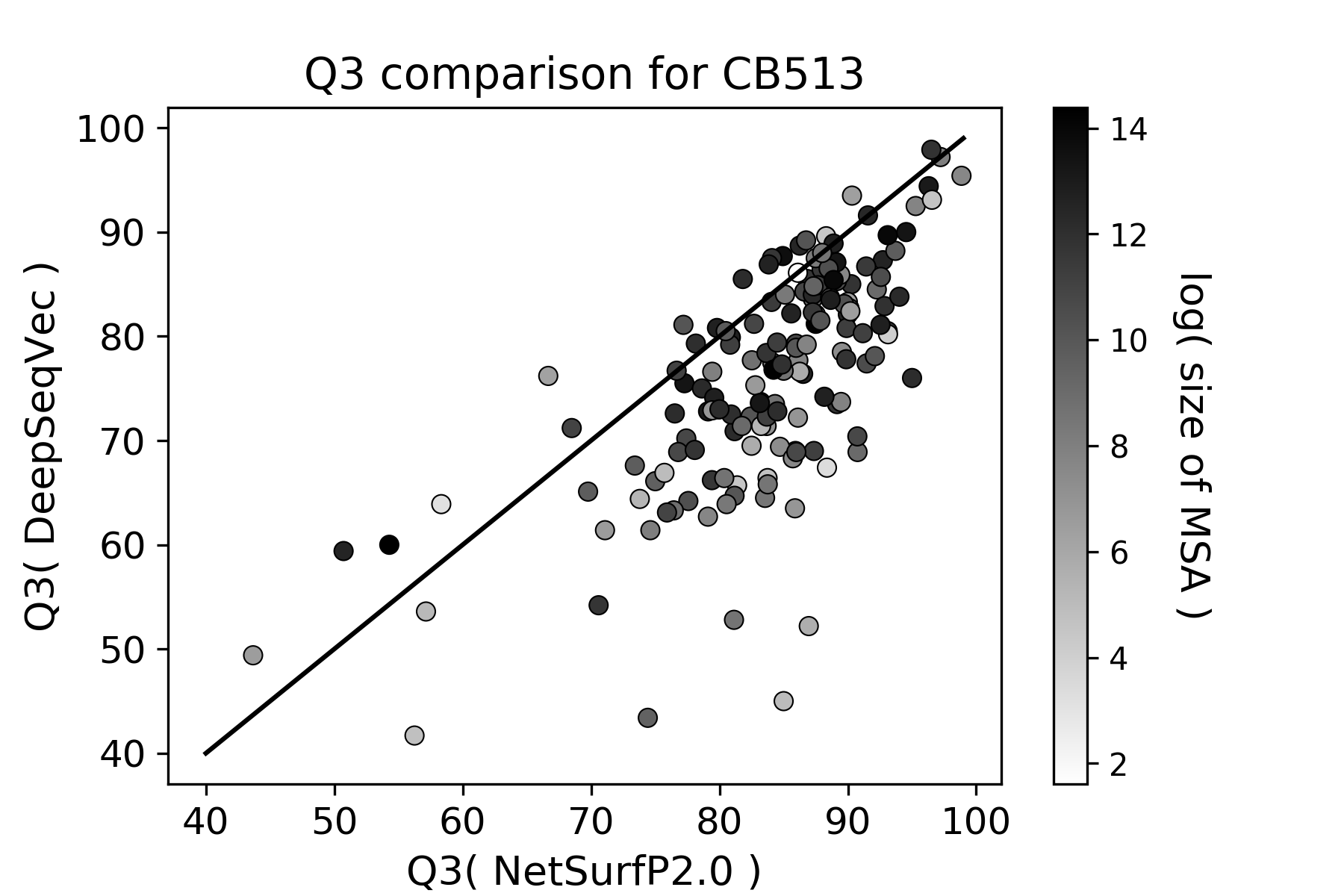


**Fig. S4: Comparison of secondary structure predictions between NetSurfP-2.0 and DeepSeqVec.** The performance of secondary structure predictions measured as accuracy in 3-states (Q3) per protein between NetSurfP-2.0 and DeepSeqVec is compared here. Proteins above the line are better predicted by DeepSeqVec (12%) while proteins below the line are better predicted by NetSurfP-2.0. Each protein is shaded based on the logarithm of the number of sequences in its MSA. For this, pre-computed MSAs were taken from ProteinNet. However, this information was only available for a subset of the proteins in our test sets (CASP12: 0%, TS115: 28%, CB513: 42%) which is why we focused our analysis on CB513.

###### Table S1: Amino acid occurrences in UniRef50 ^◊^

| Amino acid (one-letter code) | Number of occurrences | % of data | Amino acid (full name) |
| --- | --- | --- | --- |
| L | 918255239 | 9.6 | Leucine |
| A | 815091587 | 8.5 | Alanine |
| S | 721399187 | 7.5 | Serine |
| G | 651415980 | 6.8 | Glycine |
| V | 620159476 | 6.5 | Valine |
| E | 590517634 | 6.2 | Glutamic Acid |
| R | 557775181 | 5.8 | Arginine |
| T | 549632104 | 5.7 | Threonine |
| I | 530954378 | 5.5 | Isoleucine |
| D | 525972161 | 5.5 | Aspartic Acid |
| K | 494682943 | 5.2 | Lysine |
| P | 479898431 | 5.0 | Proline |
| N | 405230829 | 4.2 | Asparagine |
| F | 374988414 | 3.9 | Phenylalanine |
| Q | 374551957 | 3.9 | Glutamine |
| Y | 284178589 | 3.0 | Tyrosine |
| H | 210995340 | 2.2 | Histidine |
| M | 207346116 | 2.2 | Methionine |
| C | 135532698 | 1.4 | Cysteine |
| W | 123527183 | 1.3 | Tryptophan |
| X | 5778293 | 0.06 | Any amino acid |
| B | 3780 | 4e-5 | D or N |
| Z | 1363 | 1e-5 | E or Q |
| U | 1043 | 1e-5 | Selenocysteine |
| O | 47 | 5e-7 | Pyrrolysine |
| Total | 9577889953 | 100 |  |

^◊^ Given are the occurrences of all 20 common plus 2 rare amino acids (U and O) and 3 symbols for special cases (B means either D or N, Z means either E or Q and X means that the residue is unknown) which were used to train ELMo on all 33M proteins from UniRef50 (sorted in decreasing order).
